# Myocardial infarction impaired wall mechanics and hemodynamics in peripheral arteries

**DOI:** 10.3389/fphys.2023.1266568

**Published:** 2023-08-29

**Authors:** Qiang Xue, Hongyu Shi, Li Li, Qing Jin, Xuan Wang, Yunlong Huo

**Affiliations:** ^1^ Department of Cardiology, Yanan Hospital Affiliated to Kunming Medical University, Kunming, Yunnan, China; ^2^ Department of Cardiology, Zhongshan Hospital Wusong Branch, Fudan University, Shanghai, China; ^3^ PKU-HKUST Shenzhen-Hong Kong Institution, Shenzhen, Guangdong, China; ^4^ Institute of Mechanobiology and Medical Engineering, School of Life Sciences and Biotechnology, Shanghai Jiao Tong University, Shanghai, China

**Keywords:** myocardial infarction, wall mechanics, hemodynamics, Womersley model, peripheral artery

## Abstract

Myocardial infarction (MI) impaired both cardiac functions and peripheral arteries. The changes in normal and shear stresses in the peripheral artery wall are of importance for understanding the progression of MI-induced heart failure (HF). The aim of the study is to investigate the corresponding changes of normal and shear stresses. The coronary artery ligation was used to induce the MI in Wistar rats. The analysis of wall mechanics and hemodynamics was performed based on *in vivo* and *in vitro* measurements. Myocardial infarction increased wall stiffness in elastic carotid and muscular femoral arteries significantly albeit different changes occurred between the two vessels from 3 to 6 weeks postoperatively. Moreover, the hemodynamic analysis showed the gradually deteriorated wall shear stress, oscillatory shear index and relative residence time in the two arteries. This study probably shed light on understanding the interaction between abnormal systemic circulation and peripheral mechanics and hemodynamics during the development of MI-induced HF.

## 1 Introduction

Myocardial infarction (MI) induces ischemia that could further cause heart failure (HF) with reduced ejection fraction (HFrEF) (Humeres and Frangogiannis, 2019). Mechanical property of vessel wall and hemodynamics in peripheral arteries are important elements that affected the occurrence and progression of MI-induced HFrEF (Kassab, 2006; Huo and Li, 2022). Poelzl et al. showed that severe HF was associated with brachial artery remodeling, characterized by morphological, mechanical, and functional changes in the vessel wall (Poelzl et al., 2005). Wang et al. showed that MI resulted in the high peripheral resistance, pulse wave velocity (PWV) and augmentation index (AIx) and the low total compliance as well as a significant increase of myocardial fibers and peripheral artery wall collagens (Wang et al., 2022).

The changes in normal and shear stresses in the peripheral artery wall should be taken into account to investigate basic mechanisms for the occurrence and progression of MI-induced HFrEF (Miyoshi and Ito, 2021). Mechanical tests have been used to characterize the stress-tension response of arteries and provide sufficient data for mathematical modeling of their behavior (Humphrey and Canham, 2000; Humphrey, 2002; 2008; Huo et al., 2012; Huo et al., 2013; Lu et al., 2017; Feng et al., 2021). The Womersley model has also been applied to the analysis of pulsatile flow velocity profile, wall shear stress (WSS), oscillatory shear index (OSI) and relative residence time (RRT) in aorta and peripheral arteries (Bing et al., 2020; Li et al., 2021). Hence, the objective of the study is to investigate the changes in normal and shear stresses in peripheral artery wall during the development of MI-induced HFrEF.

Myocardial infarction was created through the ligation of the left anterior descending artery (LAD) in Wistar rats. Hemodynamic measurement and mechanical test were carried out in carotid arteries (CA) and femoral arteries (FA) *in vivo* and *in vitro*, based on which the mathematical analysis was demonstrated to compute normal and shear stresses. The significance and limitation were discussed for understanding the progression of heart failure caused by myocardial infarction.

## 2 Methods

### 2.1 Experimental measurements

#### 2.1.1 Animal preparation

**FIGURE 1:**
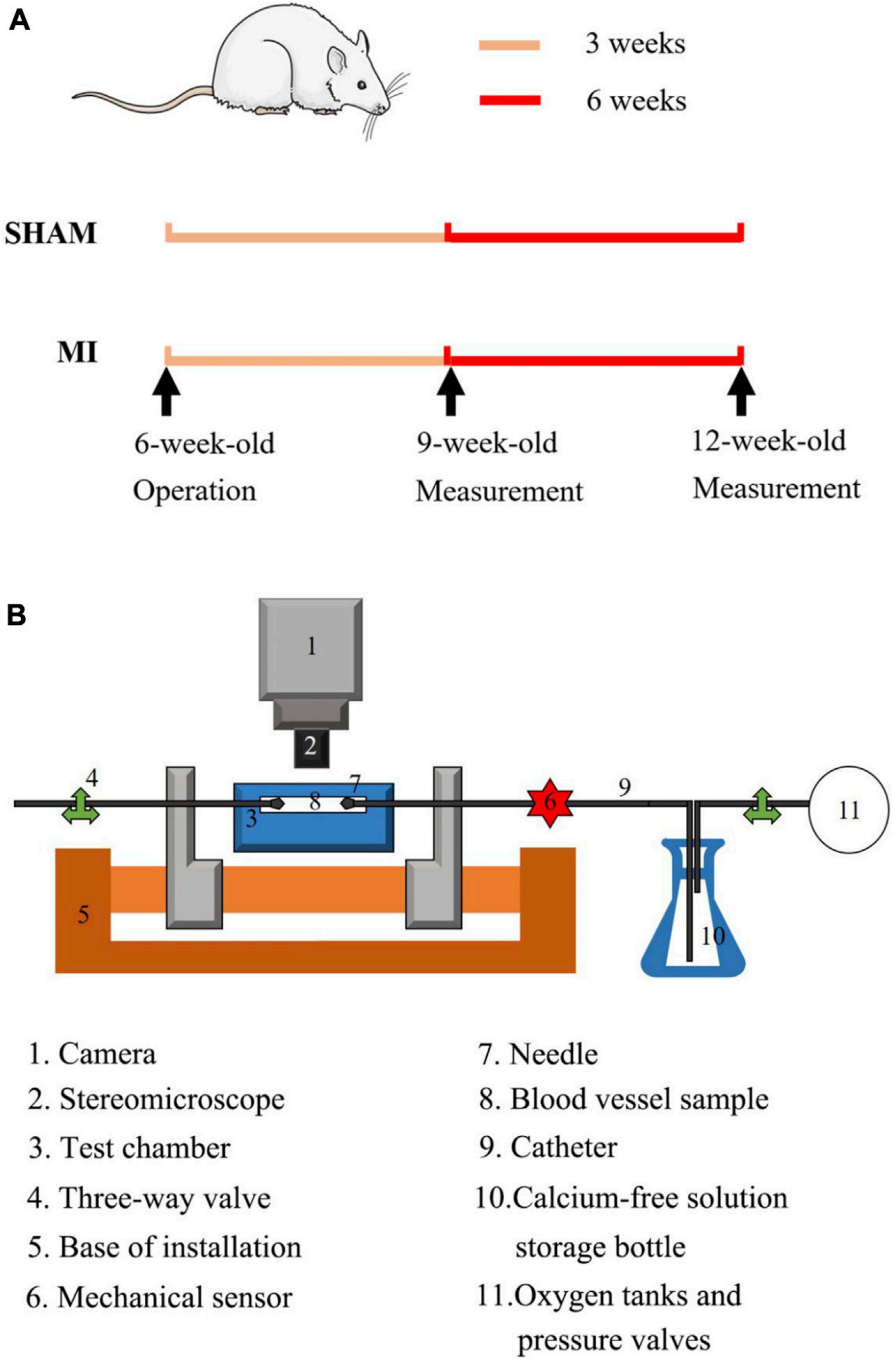
**(A)** Schematic representative of experimental protocol: echocardiographic and hemodynamic measurements were demonstrated at postoperative 3 and 6 weeks; **(B)** Overview of the utilized extension-inflation setup with inserted artery. 1. Camera; 2. Stereomicroscope; 3. Test chamber; 4. Three-way valve; 5. Base of installation; 6. Mechanical sensor; 7. Needle; 8. Blood vessel sample; 9. Catheter; 10. Calcium-free solution; 11. Oxygen tanks and pressure valves.

Six-week-old Wistar male rats (Beijing Vital River Laboratory Animal Technology Co., Ltd.) were used in the study. As shown in Figure 1A, the experimental protocol has been described in details in a previous study (Wang et al., 2022), which was approved by the Animal Care and Use Committee of Peking University, China. Here, 15 rats underwent ligation of the left anterior descending (LAD) artery of the heart as the MI group and 11 rats as the sham group. Briefly, A 7-0 suture line was placed at ∼1 mm below the left auricle appendage to close the LAD artery, which led to pale LV anterior wall and apex region. Alternatively, the suture was placed but removed in sham-operated animals. After the chest was closed, all animals were housed at standard SPF laboratory for 3 or 6 weeks after operation and free access to standard rodent chow and water. The sham and MI groups has the survival rate of 91% (*n* = 10) and 67% (*n* = 10), respectively, which were included in the following experimental measurements. The animals of MI were divided into two groups of MI-3 (*n* = 5) and MI-6 (*n* = 5) according to postoperative times (i.e., 3, 6 weeks) after the LAD ligation. Accordingly, the shams were divided into two groups of SHAM-3 (*n* = 5) and SHAM-6 (*n* = 5). All experiments were performed in accordance with the Chinese National and Peking University ethical guidelines regarding the use of animals in research, consistent with the NIH guidelines (Guide for the care and use of laboratory animals) on the protection of animals used for scientific purposes.

#### 2.1.2 Hemodynamic measurements

There is a distinct difference in microstructural and biomechanical properties between elastic and muscular arteries (Feng et al., 2021). There are packaged differences of VSMCs and elastic lamellae between elastic and muscular arteries. The media layer of elastic artery incudes many concentric elastic laminae between internal and external elastic laminae while the muscular artery only has an internal lamina. Moreover, elastic and muscular arteries have different ratios of constitutions, e.g., elastic and collagen fibers and VSMCs. Similar to previous studies (Bing et al., 2020; Li et al., 2021; Wang et al., 2022), the right elastic CA and right muscular FA were dissected in anesthetized animals before termination at postoperative 3 and 6 weeks. Perivascular flow probes (Transonic Systems Inc.; relative error of ± 2%) were mounted on the two arteries to measure flow waves. A 1.4F micromanometer-tipped catheter (Millar Instruments) was calibrated for zero pressure in 37°C saline and inserted into the two arteries to record pressure waves over 10 cardiac cycles. All data were monitored with a BIOPAC MP150.

#### 2.1.3 Mechanical tests

We carried out mechanical tests in the left CA and FA, similar to previous studies (Huo et al., 2012; Huo et al., 2013; Feng et al., 2021). Here, we chose the axial stretch ratio of 1.4 and 1.2 for the passive test using the myograph in Figure 1B At the axial stretch ratio of 1.4, the intravascular pressure increased to 15 mmHg gradually and the vessel was equilibrated for 45 min in the HEPES PSS aerated with 95% O_2_-5% CO_2_ in room temperature. Subsequently, PSS in the arteries and baths was replaced with the 37°C Ca^2+^-free Krebs solution. After equilibrating the vessels for 15 min to have full vasodilation, the transmural pressure increased from 20 to 180 mmHg by an increment of 20 mmHg. Vessel images were recorded by a CCD camera (DS-Ri2, Nikon, Resolution: 4,908 pixels × 3,264 pixels; 7.3 μm × 7.3 μm) and the changes of outer diameter were measured by the ImageJ software (NIH). Microbeads (60 μm in diameter) were used on the outer surface of vessels to mark the same place of each measurement. The passive test was repeated at the axial stretch ratio of 1.2 in the two arteries. Moreover, morphological measurements in the zero-stress state were consistent with a previous study (Feng et al., 2021).

### 2.2 Mathematical models

#### 2.2.1 Womersley analysis

Similar to a previous study (Bing et al., 2020), the equation for the pulsatile flow velocity profile across the lumen, $u\left(r,t\right)$ , is given as

$$u\left(r,t\right)=REAL\left[\frac{2Q\left(0\right)\left(R^{2}-r^{2}\right)}{\pi R^{4}}+\sum_{\omega =1}^{\infty }\frac{\frac{Q\left(\omega \right)}{\pi R^{2}}∙(1-\frac{J_{0}\left(\Lambda r/R\right)}{J_{0}\left(\Lambda \right)}}{1-\frac{2J_{1}\left(\Lambda \right)}{\Lambda J_{0}\left(\Lambda \right)}}e^{i\omega t}\right]$$ (1)

where *r* is the radial coordinate, *R* is the radius of artery, $\Lambda^{2}=i^{3}\alpha^{2}$ , $\alpha =R\sqrt{\frac{\omega \rho }{\mu }}$ , $q_{measured}\left(t\right)=Q\left(\omega \right)e^{i\omega t}$ , ω is the angular frequency after Fourier transformation, $J_{0}$ is a Bessel function of zero order and first kind, and $J_{1}$ is a Bessel function of first order and first kind. Accordingly, $\tau \left(R,t\right)$ , time-averaged WSS (TAWSS), and OSI for pulsatile blood flow can be written as:

$$\tau \left(R,t\right)=REAL\left(\frac{4\mu }{\pi R^{3}}Q\left(0\right)-\sum_{\omega =1}^{\infty }\frac{\frac{\mu Q\left(\omega \right)}{\pi R^{3}}∙\frac{{\Lambda J}_{1}\left(\Lambda \right)}{J_{0}\left(\Lambda \right)}}{1-\frac{{2J}_{1}\left(\Lambda \right)}{\Lambda J_{0}\left(\Lambda \right)}}e^{i\omega t}\right)$$ (2)

$$TAWSS=\frac{1}{T}\int_{0}^{T}\left|\tau \left(R,t\right)\right|$$ (3)

$$OSI=\frac{1}{2}\left(1-\frac{\left|\frac{1}{T}\int_{0}^{T}\tau \left(R,t\right)\right|}{\frac{1}{T}\int_{0}^{T}\left|\tau \left(R,t\right)\right|}\right)$$ (4)

The viscosity (*μ*) and density (*ρ*) were assumed to be 4.0 cp and 1.06 g/cm^3^, respectively. Moreover, RRT reflects the residence time of flow particles near the wall and is recommended as a single metric of low oscillating shear stress, which is expressed as follows:

$$RRT=\frac{1}{\left(1-2\cdot OSI\right)\cdot TAWSS}$$ (5)

#### 2.2.2 Strain energy function

The 2D passive model (Feng et al., 2021) were selected to characterize mechanical properties of the CA and FA, which can be written as:

$$W_{passive}=\frac{1}{2}C_{1}\left[e^{Q}-1\right]$$ (6)

where $Q=a_{1}E_{\theta \theta }^{2}+a_{2}E_{zz}^{2}+2a_{3}E_{\theta \theta }E_{zz}$ ; $C_{1},and\;a_{1}∼a_{3}$ are material constants; $E_{\theta \theta }$ and $E_{zz}$ are circumferential and axial Green strains, respectively; and $\lambda_{\theta }$ and $\lambda_{z}$ represent the corresponding stretch ratios. Here, $\lambda_{\theta }=\frac{l}{l_{0}}=\sqrt{{2E}_{\theta \theta }+1}\;and\;\lambda_{z}=\frac{L}{L_{0}}=\sqrt{{2E}_{zz}+1}$ , where l and $l_{0}$ are circumferential lengths in loaded and no-load states (where the vessel rings are in fully vasodilation); and L and $L_{0}$ are axial lengths in loaded and no-load states (before the vessel was cannulated to the bath). Determination of material constants have been described in details in the previous study (Feng et al., 2021). The first Piola-Kirchhoff (PK) stresses (i.e., $T_{\theta \theta }$ and $T_{zz}$ ) are computed from the 2D strain energy functions theoretically (Feng et al., 2021), which can be written as:

$$T_{\theta \theta }=\frac{\partial W_{passive}}{\partial \lambda_{\theta }},\;T_{ZZ}=\frac{\partial W_{passive}}{\partial \lambda_{z}}$$ (7)

### 2.3 Data analysis

The mean ± SD (standard deviation) values of different parameters were determined (averaged over all carotid and femoral arteries in the four groups). Two-way ANOVA (SigmaStat 3.5) was used to compare wall mechanics and hemodynamic parameters. If indicated statistically significant effect, groups were compared by the Bonferroni test for a comparison of parameters, where *p*-value < 0.05 represented the statistically significant difference.

## 3 Results

**FIGURE 2:**
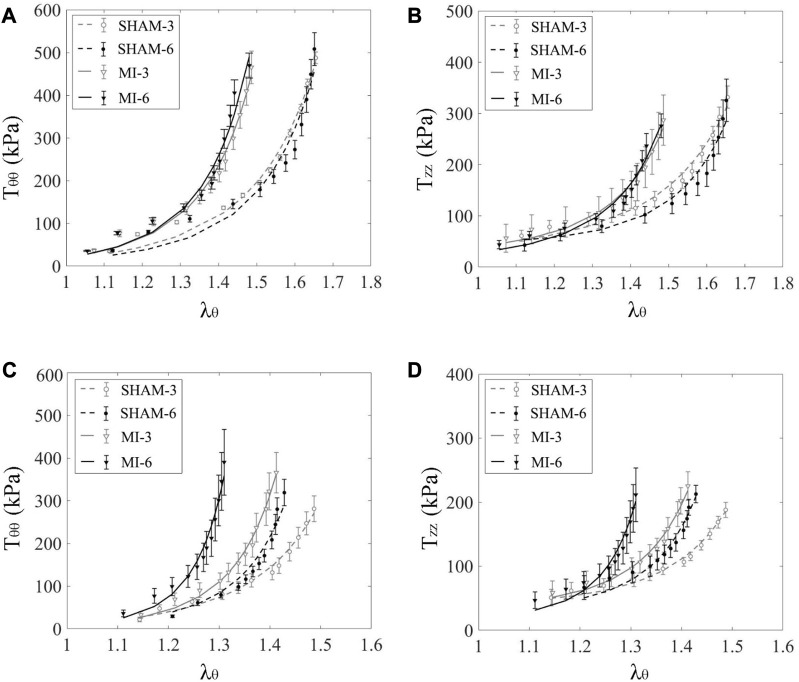
Circumferential **(A)** and axial **(B)** first PK stresses ( $T_{\theta \theta }$ and $T_{zz}$ ) as a function of circumferential stretch ratio in carotid arteries of SHAM-3 (*n* = 5), SHAM-6 (*n* = 5), MI-3 (*n* = 5) and MI-6 (*n* = 5) rats ( $\lambda_{z}$ = 1.4) (marked with error bars of SD). **(C, D)** show values in femoral arteries corresponding to **(A, B)**.

Figure 1 shows schematic representative of experimental protocol. Figures 2A, B show the circumferential and axial first PK stresses, respectively, obtained from mechanical tests in the elastic CA of SHAM-3, SHAM-6, MI-3, and MI-6 groups. Accordingly, Figure 2B shows the first PK stresses in the muscular FA of the four groups. The first PK stress in the circumferential direction is higher than that in the axial direction ( $T_{\theta \theta }≅2∙T_{zz}$ ). Myocardial infarction results in a significant increase of circumferential and axial stresses. Table 1 lists material constants by the optimal fit of all measured data in CA and FA of each group using the 2D passive Fung model. The stress-stretch curves have an approximately linear relationship in the physiological range of 60–140 mmHg, the slope of which is determined by the linear least square fit. The high slope denotes the high stiffness of vessel wall. Carotid arteries have similar wall stiffnesses between SHAM-3 and SHAM-6 groups and between MI-3 and MI-6 groups. Femoral arteries have higher values of wall stiffness in the MI-6 and SHAM-6 groups than the MI-3 and SHAM-3 groups, respectively, which shows an age-related increase of wall stiffness in the muscular FA.

**TABLE 1:** Material constants by the optimal fit of all measured data in the carotid artery and femoral artery of SHAM-3, SHAM-6, MI-3 and MI-6 groups.

Vessels	Groups	$a_{1}$	$a_{2}$	$a_{3}$	$C_{1}$	$R_{T\theta \theta }^{2}$	$R_{Tzz}^{2}$
CA	SHAM-3	1.14	1.05	0.55	48.24	0.95	0.97
	SHAM-6	1.42	1.06	0.79	24.53	0.95	0.96
	MI-3	2.50	1.52	0.81	28.48	0.95	0.95
	MI-6	2.46	1.15	1.06	29.26	0.97	0.96
FA	SHAM-3	2.56	1.97	0.50	19.25	0.94	0.95
	SHAM-6	5.90	4.37	0.10	4.72	0.95	0.95
	MI-3	5.63	3.40	0.32	8.41	0.95	0.94
	MI-6	13.77	4.97	0.46	2.53	0.94	0.96

*R* ^2^ expresses the square of correlation coefficients between experimental measurements and optimal fits, the higher value of which denotes the better fit in the range of 0–1.

**FIGURE 3:**
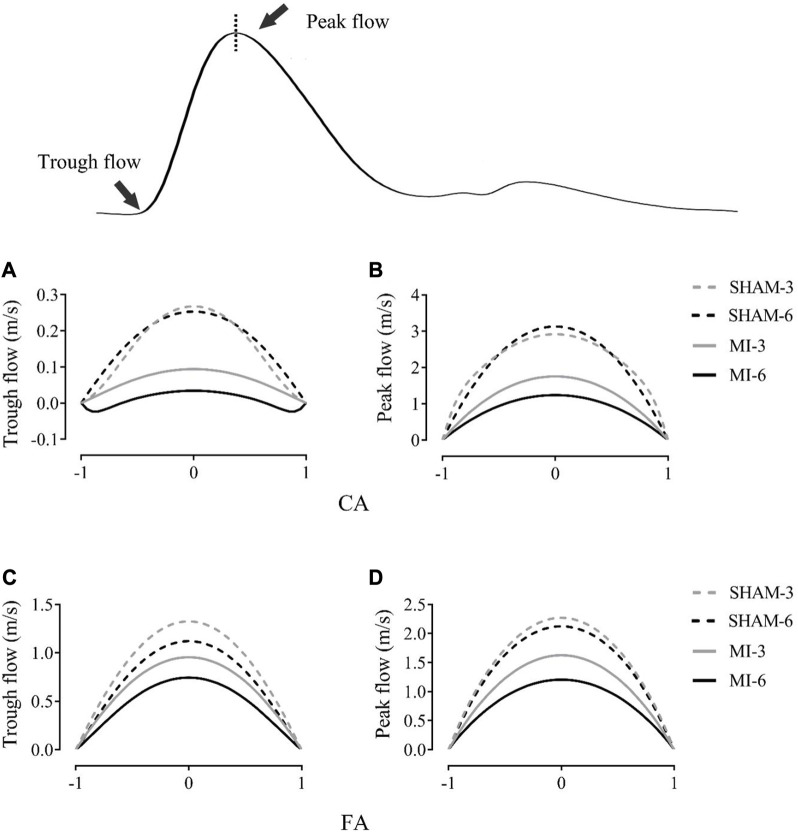
Flow velocity profiles (m/s) in the carotid artery at time instances of trough **(A)** and peak **(B)** flows. **(C, D)** show values in femoral arteries corresponding to **(A, B)**.

**FIGURE 4:**
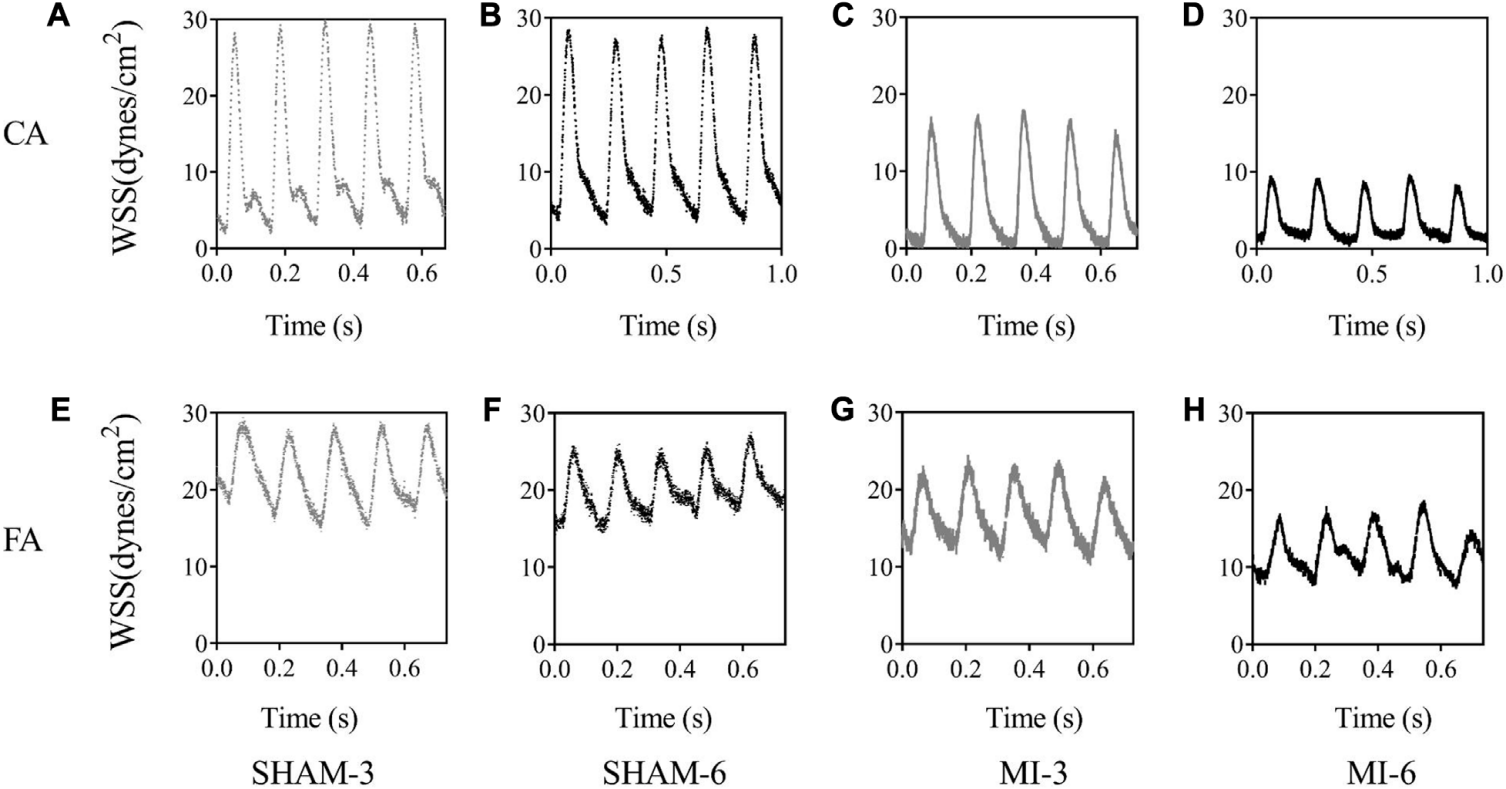
Transient WSS waves (unit: dynes/cm^2^) in the carotid artery of SHAM-3 **(A)**, SHAM-6 **(B)**, MI-3 **(C)** and MI-6 **(D)**. **(E–H)** show values in femoral arteries corresponding to **(A–D)**.

Figure 3 shows flow velocity profiles in CA and FA of SHAM-3, SHAM-6, MI-3, and MI-6 groups at time instances of trough and peak flows. Figure 4 shows transient WSS waves (unit: dynes/cm^2^) in CA and FA of the 4 groups. The transient WSS waves significantly decreases in the MI-3 group and has the lowest time-averaged value and amplitude in the MI-6 group. The trough flow of CA in MI-6 group shows the strong reversal flow near the wall. Accordingly, Table 2 lists mean ± SD values of TAWSS, OSI, and RRT, which show no statistical difference between SHAM-3 and SHAM-6 groups. Myocardial infarction causes low TAWSS and high OSI and RRT. The MI-6 group has the lowest TAWSS and the highest OSI and RRT than other groups.

**TABLE 2:** TAWSS, OSI, and RRT in the carotid artery and femoral artery of SHAM-3, SHAM-6, MI-3 and MI-6 groups.

Groups		SHAM-3	SHAM-6	MI-3	MI-6
CA	TAWSS (dynes/cm^2^)	7.55 ± 1.78^*^	6.90 ± 1.34^#^	4.72 ± 1.14	3.87 ± 0.73
	OSI ( × 10^−3^)	0.00 ± 0.00^*^	0.02 ± 0.03^#^	0.53 ± 0.85	2.58 ± 2.32^◆^
	RRT	1.39 ± 0.26	1.27 ± 0.39	1.89 ± 0.63	2.76 ± 0.52
FA	TAWSS (dynes/cm^2^)	25.4 ± 5.25^*^	20.5 ± 0.77^#^	14.0 ± 1.97	10.1 ± 5.3
	OSI ( × 10^−3^)	0.00 ± 0.00	0.00 ± 0.00	0.00 ± 0.00	0.61 ± 0.00^◆^
	RRT	0.47 ± 0.09	0.49 ± 0.02^#^	0.73 ± 0.12	1.01 ± 0.64

Values are means ± SD. *p < 0.05, SHAM-3, vs. MI-3; #*p* < 0.05, SHAM-6, vs. MI-6; ◆*p* < 0.05, MI-6, vs. MI-3.

## 4 Discussion

This study determined the first PK stresses (i.e., $T_{\theta \theta }$ and $T_{zz}$ ), WSS, OSI and RRT in CA and FA in shams and MI rats at postoperative time instances. The main findings are reported as: 1) myocardial infarction impaired both normal and shear stresses as well as other hemodynamic parameters; 2) the MI-6 group had the worst environment of wall mechanics and hemodynamics in CA and FA; and 3) the elastic CA and the muscular FA had different changes during the progression of MI-induced HF.

Myocardial infarction impaired the LV systolic function (Thygesen et al., 2007; Lu et al., 2020; Jenca et al., 2021), which induced a gradual decrease of cardiac output (CO) and mean aortic pressure (MAP) with time (Wang et al., 2022). The reduced CO and MAP deteriorated the systemic circulation, e.g., an increase of total peripheral resistance and a decrease of total compliance. Here, we demonstrated the analysis of wall mechanics and hemodynamics in peripheral arteries. In both elastic CA and muscular FA, MI resulted in a significant increase of wall stiffness, consistent with previous studies (Hirai et al., 1989; Wang et al., 2022). There was no statistical difference of wall stiffness in the CA between SHAM-3 and SHAM-6 groups and between MI-3 and MI-6 groups. There was, however, a significant increase of wall stiffness in the FA of sham or MI groups from 3 to 6 weeks postoperatively. We have shown the relatively unchanged wall stiffness in healthy carotid arteries between juvenile and adult rats, but an increase of femoral arterial stiffness in the adult rats (Feng et al., 2021), consistent with the present observations in the sham groups. On the other hand, we showed an increase of collagen type III and I in both CA and FA of MI rats from 3 to 6 weeks postoperatively (Wang et al., 2022). It is known that a distinct difference in microstructural and biomechanical properties occurred between muscular and elastic arteries (Wagner and Humphrey, 2011; Feng et al., 2021; Murtada et al., 2021; Wang et al., 2022). The carotid artery had internal and external elastic laminae plus many concentric elastic laminae within the media (Feng et al., 2021), which cut down the effect of the increased collagen. Hence, there was the relatively unchanged carotid arterial stiffness between MI-6 and MI-3 groups. On the other hand, the increased collagen within the media of the MI-6 group induced a significant increase of wall stiffness than the MI-3 group because the femoral artery had a prominent internal elastic lamina only.

The abnormal hemodynamic parameters, i.e., low WSS and high OSI and RRT, were known to impair the endothelial cells of blood vessels (Chen et al., 2016; Huang et al., 2016; Fan et al., 2019). The Womersley analysis showed that TAWSS in CA and FA of the MI groups was >35% lower and RRT was >35% higher than the sham groups. There was a significant increase of OSI in the MI groups from 3 to 6 weeks postoperatively despite negligible OSI in the sham groups. Myocardial infarction gradually worsened the hemodynamic environment in both CA and FA, such as the reduction of WSS, the increase of OSI, and the prolongation of RRT. On the other hand, the Womersley analysis showed the velocity profiles in CA and FA at time instances of trough and peak flows. Myocardial infarction changed parabolic profiles to blunt profiles as well as induced reversal flows near the vessel wall in the MI-6 group. The MI-induced abnormal changes can result in the endothelial dysfunction, monocyte deposition, microemboli formation, SMC proliferation in peripheral arteries (Davies, 2009; Chiu and Chien, 2011; Green et al., 2017; Han et al., 2018), which requires further investigations.

The interplay of cardiac dysfunctions and total impairments in systemic circulation was known to accelerate the progression of MI-induced HF (Wang et al., 2022). Here, the analysis of vessel wall mechanics and hemodynamics demonstrates that the increased normal stress and the decreased shear stress as well as other abnormal parameters impaired vessel wall, which contributed to the development of MI-induced HF. Hence, the detrimental changes in peripheral arteries should be considered to study the MI-induced HF.

## 5 Critiques of the study

This study only carried out passive mechanical tests in both CA and FA during the development of MI-induced HF. The active mechanical tests should be performed for understanding the interplay of MI and peripheral vessel wall mechanics. A three-dimensional computational fluid dynamic model, coupled with morphometric data of peripheral arteries, should be used to investigate abnormal hemodynamic parameters in details. The relevant molecular and cellular mechanisms should be demonstrated in the following studies.

## 6 Conclusion

The analysis of wall mechanics showed an increase of MI-induced wall stiffness, which had different changes with time between elastic CA and muscular FA. The hemodynamic analysis demonstrated the gradually worsened WSS, OSI and RRT in peripheral arteries during the progression of MI-induced HF. Both normal and shear stresses are of importance for understanding the MI-induced HF.

## Data Availability

The raw data supporting the conclusion of this article will be made available by the authors, without undue reservation.
